# Recent Advances in AS101 and Tellurium-Based Nanoplatforms for Immunomodulation, Cancer Therapy, Antimicrobial Activity, and Toxicological Assessment

**DOI:** 10.1021/acsomega.6c03522

**Published:** 2026-07-15

**Authors:** Ana Paula dos Santos Cardoso, Alcindo A. Dos Santos, Gabriel Lima Barros de Araujo

**Affiliations:** † Department of Pharmacy, School of Pharmaceutical Sciences, 67781 University of São Paulo, Av. Professor Lineu Prestes, 580 Cidade Universitária, São Paulo CEP 05508-000, SP, Brazil; ‡ Institute of Chemistry, University of São Paulo (USP), Av. Professor Lineu Prestes, 748 Cidade Universitária, São Paulo CEP 05508-000, SP, Brazil

## Abstract

Tellurium (Te), a metalloid historically associated with toxicity, has recently gained attention for its potential biomedical applications through advances in AS101-based pharmacology and tellurium nanotechnology. This review discusses the evolution of tellurium research from the immunomodulatory compound AS101 [ammonium trichloro­(dioxoethylene-O,O′)­tellurate] to modern tellurium nanoparticles (TeNPs) and nanoheterojunctions. Experimental studies indicate that AS101 modulates cytokine signaling, inhibits integrin activation, and exerts immunomodulatory effects through thiol redox regulation. Preclinical evidence also suggests neuroprotective and anti-inflammatory effects in animal models of neurodegenerative and mood-related disorders. In parallel, TeNPs and Te-based heterostructures have been investigated in vitro and in animal models for applications in photothermal therapy (PTT), radiosensitization, and ROS-mediated anticancer activity. In addition, TeNPs demonstrated antimicrobial and antibiofilm effects against multidrug-resistant pathogens, including methicillin-resistant *Staphylococcus aureus* (MRSA). The review also highlights recent advances in tellurium speciation analysis, metabolism, and toxicity assessment using approaches such as LC-ICP-MS. Overall, current findings support tellurium-based compounds and nanoplatforms as emerging candidates for biomedical applications, although further pharmacokinetic, toxicological, and clinical studies remain necessary to establish their long-term safety and therapeutic efficacy.

## Graphical abstract

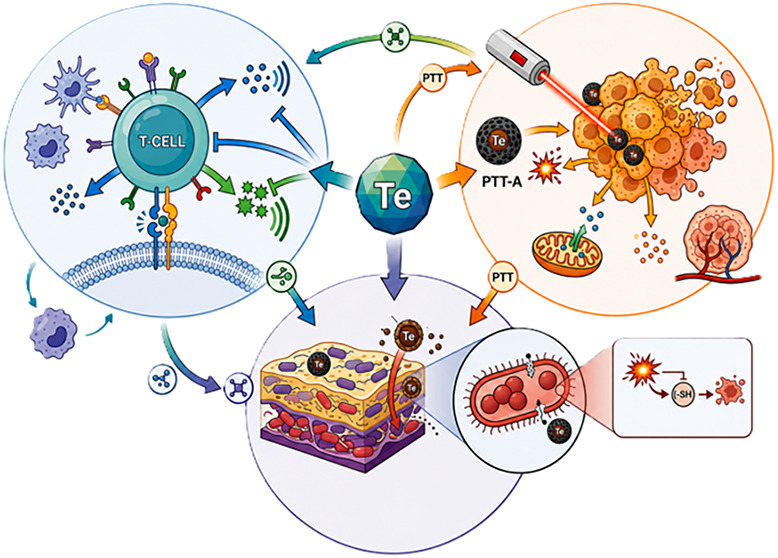

## 1 Introduction

Tellurium (Te), a chalcogen group element, was historically neglected in biomedicine due to its perception as a toxic and nonessential trace element.^1,2^ However, its chemistry offers unique reactivity, particularly its high affinity for thiol groups, allowing modulation of fundamental biological processes such as cellular redox balance, cytokine regulation, integrin signaling, and enzymatic activity.^1,2^ Therapeutic interest in this metalloid was catalyzed by the development of the tellurium-based coordination compound AS101, which became the principal proof-of-concept demonstrating that appropriately designed tellurium compounds can exhibit favorable safety profiles and significant biological activity.^1,2^ As a potent immunomodulator, AS101 modulates cytokine networks, inhibits integrin-mediated signaling pathways, and exhibits antitumor activity in several experimental cancer models.^1,3,4^ Its therapeutic versatility also extends to the central nervous system, where AS101 increases brain-derived neurotrophic factor (BDNF) levels and produces neuroprotective, anxiolytic, and antidepressant-like effects in preclinical models, suggesting potential applications in neurodegenerative and neuropsychiatric disorders.^5,6^ Additional organotellurium derivatives, such as RF-07, further reinforce this potential by protecting neuronal tissue and reducing seizure activity in experimental epilepsy models through inhibition of cysteine proteases and caspase-dependent pathways.^7^

In parallel, advances in nanotechnology have introduced tellurium nanoparticles (TeNPs) as promising platforms capable of overcoming some limitations associated with small-molecule tellurium compounds, including instability and restricted bioavailability. In oncology, TeNPs have demonstrated selective anticancer activity in vitro through mechanisms involving ROS-mediated apoptosis, calcium signaling dysregulation, and inhibition of cancer-cell migration.^8^ More sophisticated Te-based nanostructures, including Te–Se nanoheterojunctions and tellurium-driven heterostructures, have shown remarkable efficacy in photothermal and radiophotothermal therapies by combining efficient light-to-heat conversion with immune activation and tumor growth suppression in preclinical models.^9,10^ Nevertheless, while nanostructuring may improve therapeutic performance, important challenges remain regarding long-term biodistribution, biodegradation, tissue accumulation, and systemic safety, which continue to require comprehensive investigation before clinical translation.

In the field of infectiology, Te-based nanomaterials have emerged as promising alternatives in the fight against antimicrobial resistance. Several tellurium-containing nanoplatforms exhibit antibacterial activity against both Gram-positive and Gram-negative pathogens through mechanisms involving ROS generation, membrane disruption, and inhibition of biofilm formation.^11,12^ Hybrid tellurium–lignin nanoparticles and gallic acid-functionalized TeNPs have demonstrated strong antimicrobial efficacy while maintaining relatively low toxicity toward mammalian cells under experimental conditions, highlighting their potential as next-generation antimicrobial agents.^11,12^ However, most available evidence remains limited to in vitro studies, and further investigation is required to establish their safety, efficacy, and translational potential in clinical or veterinary settings.

This Review compiles current advances in the therapeutic applications of tellurium, ranging from the molecular mechanisms underlying AS101-mediated immunomodulation to emerging nanotechnological approaches. Particular emphasis is placed on critically evaluating both the therapeutic opportunities and the remaining challenges associated with tellurium-based compounds and nanomaterials, including efficacy, toxicity, biodistribution, and clinical translation. Recent reviews have further emphasized the growing interest in organotellurium chemistry, particularly regarding the synthesis of novel derivatives with antioxidant and anticancer properties, reinforcing the therapeutic potential of tellurium-based compounds.^13^

## 2 The AS101 Paradigm: Chemical Foundations and Mechanisms of Immunomodulation

AS101 [ammonium trichloro­(dioxoethylene-O,O’)­tellurate] is considered the prototypical tellurium-based medicinal compound and remains the only tellurium derivative reported to have advanced to Phase II clinical evaluation.^2,14^ Its structure consists of a Te­(IV) center arranged in a distorted square-pyramidal geometry, coordinated to three chloride ligands and a bidentate ethylene glycol moiety.^2^

The biological activity of AS101 has traditionally been attributed to the high affinity of the Te­(IV) center for thiol-containing biomolecules, particularly cysteine residues present in enzymes and cell-surface proteins.^1,2^ However, the chemical stability of AS101 in aqueous environments has been re-evaluated in recent years. Silberman et al. demonstrated that AS101 undergoes rapid ligand-substitution reactions and hydrolysis under physiological aqueous conditions, yielding the tellurium­(IV) oxide species TeOCl_3_^–^ as the predominant product.^15^ Consistent with these findings, Princival et al. confirmed that AS101 is readily converted into hydrolyzed species in aqueous media, whereas several organotellurium derivatives display substantially greater hydrolytic stability.^16^ These observations suggest that at least part of the biological activity attributed to AS101 may involve its hydrolysis products and their interactions with protein thiol groups, an aspect that is important for understanding its pharmacodynamics and for the rational design of new tellurium-based therapeutics.

## 3 Molecular Mechanisms and Therapeutic Activities of AS101

AS101 represents the prototypical tellurium-based therapeutic agent and provides a framework for understanding the biological effects of tellurium compounds. Through redox interactions with protein thiols, AS101 modulates multiple cellular pathways involved in immune regulation, cell signaling, and tissue protection. The following sections discuss the principal molecular mechanisms responsible for their diverse pharmacological activities.

### 3.1 Thiol Redox Dynamics and Thiol Group Selectivity

The fundamental basis of AS101 pharmacodynamics lies in the selective redox reactivity of Te­(IV) species toward protein thiol groups (-SH), particularly catalytically active or spatially vicinal cysteine residues.^2,15^ Unlike many heavy metals that form irreversible covalent adducts associated with nonspecific toxicity, AS101-derived Te­(IV) species exhibit preferential and potentially reversible interactions with biologically accessible thiols, resulting in redox modulation rather than indiscriminate protein damage.^2,17^

Recent mechanistic evidence suggests that AS101 behaves as a prodrug rather than as the direct reactive species. Under physiological aqueous conditions, including phosphate-buffered saline and saline solutions, AS101 rapidly undergoes hydrolysis and ligand-substitution reactions, yielding predominantly the tellurium­(IV) oxychloride species [TeOCl_3_^–^].^2,15,16^ Consequently, the biologically active Te­(IV) species that interacts with proteins is likely generated prior to thiol binding rather than corresponding to the intact parent compound.^15^ A schematic representation of the proposed hydrolysis and thiol-interaction pathway is presented in Figure 1.^2^

**Figure 1:**
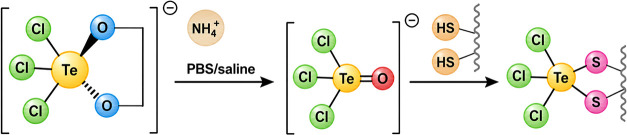
Proposed mechanism of thiol redox modulation mediated by hydrolyzed AS101-derived tellurium­(IV) species. Under physiological aqueous conditions (PBS/saline), AS101 undergoes hydrolysis and ligand exchange reactions, generating reactive tellurium oxychloride species, predominantly [TeOCl_3_^–^]. These hydrolyzed Te­(IV) species can reversibly coordinate with accessible cysteine thiol groups in proteins, forming transient Te–S intermediates. Subsequent thiol oxidation may promote disulfide bond formation and redox-dependent conformational or functional alterations in target proteins.

The proposed mechanism involves the reversible coordination of hydrolyzed Te­(IV) species to cysteine thiol/thiolate groups in target proteins, forming transient Te–S intermediates.^2,15,16^ These interactions may promote thiol oxidation and subsequent disulfide bond formation between vicinal cysteine residues, accompanied by reduction of the Te­(IV) center and/or formation of intermediate tellurium–thiol complexes.^2,17,18^ Such redox modulation functions as a molecular switch capable of inducing conformational alterations or catalytic inactivation of proteins that depend on reduced cysteine residues at their active sites, including cysteine proteases and caspases.^2,7,18^

### 3.2 Immunomodulation and Cytokine Cascade

The primary effect of AS101 lies in the induction of peripheral blood mononuclear cell (PBMC) proliferation and the promotion of immune response polarization toward a Th1-associated profile.^1,19^ Acting as a potent immunomodulator, the compound enhances the production of cytokines involved in cellular immunity, particularly interleukin-2 (IL-2) and interferon-γ (IFN-γ), which are essential mediators of antitumor and antiviral responses.^1^ Increased expression of IL-2 and its receptor (IL-2R) is considered a hallmark of AS101 activity.^19^ These effects contribute to the restoration of immune competence in immunocompromised individuals and in patients undergoing chemotherapy.^1^

In parallel with its stimulatory effects on cellular immunity, one of the most clinically relevant mechanisms of AS101 is its ability to inhibit the production of interleukin-10 (IL-10), a cytokine frequently associated with tumor immune evasion.^1,4^ By suppressing IL-10-mediated signaling, AS101 counteracts tumor-induced immunosuppression and contributes to the maintenance of effective antitumor immune responses. Recent studies have further demonstrated that tellurium-based compounds such as AS101 can modulate pathways involved in immune checkpoint regulation and tumor immune escape, reinforcing their immunotherapeutic potential.^4^

Beyond the oncological context, AS101 demonstrates remarkable versatility in autoimmune disorders, highlighting its context-dependent immunomodulatory activity. While the compound promotes Th1-associated responses and enhances IL-2 production under conditions of immunosuppression or impaired immune surveillance, its effects in autoimmune diseases are primarily related to the restoration of immune homeostasis rather than generalized immune stimulation.^14^ In these settings, AS101 selectively modulates the balance among Th1, Th2, and Th17 lymphocyte subsets, suppressing pathogenic inflammatory pathways while preserving protective immune functions. In particular, the compound has been shown to reduce the production of pro-inflammatory cytokines such as IL-17, enhance regulatory immune mechanisms, and attenuate inflammatory cell infiltration.^14^ These properties have supported its investigation in autoimmune diseases such as psoriasis and multiple sclerosis, in which dysregulated Th17 responses play a central pathogenic role. Furthermore, the anti-inflammatory effects of AS101 are reinforced by its redox-mediated inhibition of integrins, particularly VLA-4 (α4β1), which limits leukocyte adhesion and transendothelial migration into inflamed tissues, thereby reducing chronic tissue damage and disease progression.^4,14^

### 3.3 Action on Integrins and Metastasis

The understanding of the therapeutic mechanisms of AS101 has evolved significantly beyond cytokine modulation and currently includes the regulation of cell adhesion, migration, and tumor immune evasion. Experimental studies have demonstrated that AS101 acts as a potent redox-dependent inhibitor of specific integrins, particularly α4β1 integrin (very late antigen-4, VLA-4), through selective modification of critical extracellular cysteine residues rather than by reducing integrin surface expression.^4,14^ The interaction of Te­(IV) species with vicinal thiols in the extracellular domain of the α4 chain induces conformational alterations that prevent the transition of VLA-4 to its high-affinity state, thereby impairing ligand binding and downstream signaling.^4^

This inhibition of integrin activity has important implications for tumor progression and metastasis. VLA-4 mediates the interaction of malignant cells with vascular cell adhesion molecule-1 (VCAM-1) and extracellular matrix components, processes that facilitate adhesion, migration, and tissue colonization. By suppressing VLA-4 activity, AS101 interferes with these adhesive interactions and may reduce the capacity of tumor cells to establish metastatic niches.^4,14^ More recently, inhibition of VLA-4 signaling by AS101 was shown to decrease activation of the PI3K/Akt/NF-κB pathway and consequently reduce programmed death-ligand 1 (PD-L1) expression in tumor cells. This effect increased CD8+ T-cell infiltration and enhanced the antitumor efficacy of anti-PD-1 immunotherapy in preclinical melanoma models, suggesting that tellurium compounds may function as adjuvants to immune checkpoint blockade therapies.^4^

In dermatological oncology, AS101 has shown promising activity against mycosis fungoides, the most common form of cutaneous T-cell lymphoma. In a xenograft model and in cultured malignant T-cell lines, AS101 delayed tumor growth and induced apoptosis through mechanisms associated with increased intracellular reactive oxygen species (ROS) production, mitochondrial membrane depolarization, and activation of caspase-9 and caspase-3.^3^ Importantly, ROS scavenging significantly reduced apoptosis, indicating that oxidative stress plays a central role in the antitumor activity of AS101 in this model.^3^ In addition to its direct antitumor effects, AS101 has consistently exhibited a favorable safety profile in preclinical and clinical studies, with limited systemic toxicity and no significant myelosuppression reported at therapeutically relevant doses.^4,14^

Beyond AS101 itself, novel organotellurium derivatives continue to emerge as promising therapeutic candidates. Tripathi et al. demonstrated that a cyclic zwitterionic organotellurolate (IV) compound exhibited significant cytotoxicity against MCF-7 breast cancer cells while maintaining good biocompatibility toward fibroblasts and showing antibacterial activity, highlighting the versatility of organotellurium scaffolds for biomedical applications.^20^

### 3.4 Neuroprotection and Modulation of the Central Nervous System (CNS)

The potential application of AS101 in neuroscience represents an emerging area of investigation. Experimental studies have demonstrated beneficial effects of the compound in animal models of depression- and anxiety-like behaviors. Chronic administration of AS101 improved behavioral outcomes in both chronic mild stress and genetically predisposed depressive models, reducing anhedonia- and anxiety-related phenotypes.^5,6^ These behavioral effects were accompanied by increased levels of brain-derived neurotrophic factor (BDNF) in the hippocampus and other brain regions, suggesting that modulation of neurotrophic signaling may contribute to the therapeutic actions of AS101.^5,6^

Beyond their effects on mood-related behaviors, tellurium­(IV) compounds have demonstrated neuroprotective properties associated with the regulation of apoptotic pathways. Experimental evidence indicates that compounds such as AS101 and related organotellurium derivatives inhibit cysteine-dependent caspases, including caspase-1, caspase-3, and caspase-8, thereby attenuating apoptosis-associated neuronal damage.^7^ In a pilocarpine-induced status epilepticus model, inhibition of these caspases was associated with anticonvulsant and neuroprotective effects, supporting the hypothesis that modulation of apoptotic signaling contributes to the biological activity of tellurium compounds in the nervous system.^7^

Additional studies have suggested that AS101 may exert anti-inflammatory and neuroprotective actions through redox modulation of thiol-containing proteins and regulation of cytokine-associated pathways. Previous investigations reported inhibition of IL-1β- and caspase-dependent signaling pathways as well as neuroprotective effects in experimental models of neurological injury.^7,17^ Furthermore, reductions in circulating corticosterone levels observed in stress-related models may contribute to the stabilization of neuroendocrine responses associated with chronic stress.^5^ Collectively, these findings indicate that AS101 and related tellurium compounds possess multifaceted biological activities that warrant further investigation as potential neuroprotective and neuromodulatory agents.

## 4 Nanotechnology and Modern Antitumor Therapies

The transition of tellurium applications from small organic molecules to the nanoscale represents a leap in precision oncology. Tellurium nanoparticles overcome the limitations of solubility and metabolic instability, allowing the metalloid to act as a theranostic agent. Their high electron density enables visualization via computed tomography, while their unique electronic properties facilitate combined physical and chemical therapies. Representative oncological applications of tellurium nanoplatforms are summarized in Figure 2.

**Figure 2:**
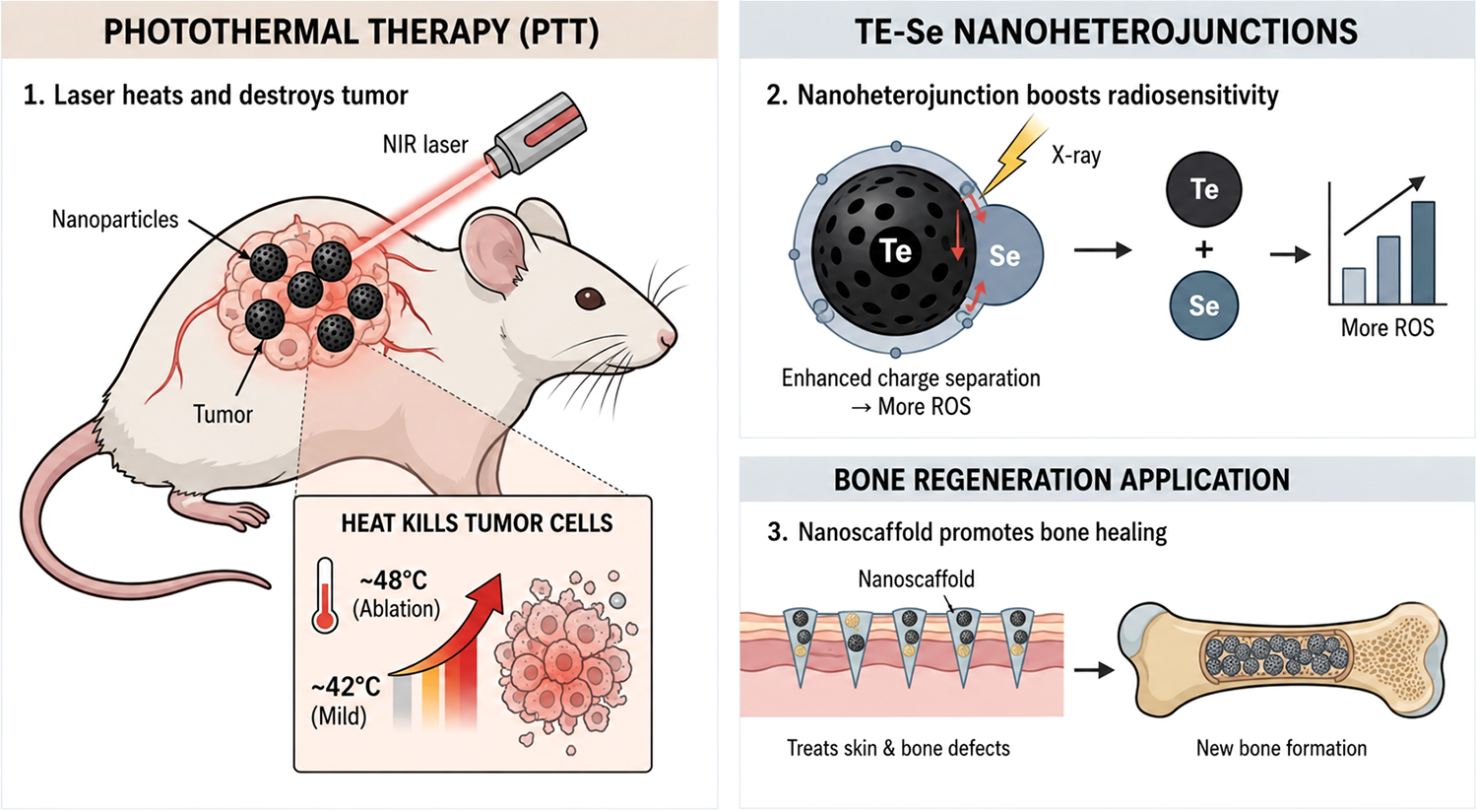
Applications of TeNPs in oncology. (1) Photothermal ablation and selective apoptosis in solid tumors under 808 nm NIR laser. (2) Enhanced radiosensitization and ROS generation by Te–Se nanoheterojunctions under X-ray irradiation. (3) Synergistic melanoma treatment via dissolvable microneedles (TeNPs + Fucoidan) and bone tissue regeneration using Te-doped bioactive glass (Te-MBG) scaffolds.

### 4.1 Advanced Phototherapy: The Power of Near-Infrared (NIR)

Recent reviews have highlighted the rapid expansion of tellurium nanomaterials in photothermal and photodynamic cancer therapy owing to their strong NIR absorption, favorable photothermal conversion, and versatile surface functionalization strategies.^21^

Elemental tellurium is a narrow-bandgap semiconductor (∼0.35 eV), a characteristic that enables strong absorption in the near-infrared (NIR) region and efficient conversion of light into thermal energy. This property has been widely explored in photothermal therapy (PTT), where specific tellurium nanostructures have demonstrated remarkable photothermal performance. For example, PEGylated ultrathin tellurium nanosheets exhibited a photothermal conversion efficiency of approximately 55% under 808 nm laser irradiation, highlighting their potential as effective photothermal agents.^22^ Upon NIR laser exposure, these nanomaterials generate localized hyperthermia capable of inducing tumor cell death while minimizing damage to surrounding healthy tissues.^22^

The evolution of crystal engineering has enabled the development of tellurium–selenium (Te–Se) nanoheterojunctions for synergistic radio-phototherapy applications. In these structures, the interface between Te and Se promotes more efficient separation of photogenerated electron–hole pairs, reducing charge recombination and enhancing reactive oxygen species (ROS) generation.^9^ This architecture significantly increases tumor radiosensitivity and has demonstrated near-complete tumor suppression in preclinical models of lung carcinoma and hepatocellular carcinoma when combined with reduced radiation and laser doses.^9^ Furthermore, treatment with Te–Se nanoheterojunctions was associated with extensive tumor cell apoptosis and modulation of the tumor microenvironment, supporting their potential as multifunctional therapeutic platforms.^9^

Complementing this advancement, the design of complex heterostructures such as BTe–Pd-Au (tellurium nanorods incorporating palladium and gold) represents a sophisticated strategy for multimodal cancer therapy.^10^ In these systems, the incorporation of palladium and gold promotes enhanced separation of photogenerated charge carriers, increases ROS generation, and improves both radiosensitization and photothermal performance.^10^ The gold core and palladium shell additionally contribute to radiotherapeutic enhancement due to their high atomic numbers, while the tellurium framework preserves the photothermal functionality.^10^ Beyond direct tumor destruction, these heterostructures have been shown to stimulate antitumor immune responses through the release of tumor-associated antigens and damage-associated molecular patterns (DAMPs), promoting dendritic cell maturation, increasing cytotoxic T-lymphocyte (CD8+) infiltration, and reducing immunosuppressive tumor-associated macrophages.^10^ In murine colon cancer models, these combined effects contributed to effective suppression of primary tumors while simultaneously activating systemic antitumor immune responses.^10^

### 4.2 Selective Cytotoxicity: Oxidative Stress and Mitochondrial Calcium Overload

Unlike inert nanocarriers, certain tellurium nanomaterials behave as biologically active agents that directly interact with the redox machinery of tumor cells. An illustrative example is provided by tellurium nanowires (TeNWs), which have been described as inorganic nanoprodrugs. In the tumor microenvironment, elevated concentrations of hydrogen peroxide (H_2_O_2_) oxidize elemental tellurium (Te^0^), generating tellurate species (TeO_6_^6–^). This reaction promotes enhanced reactive oxygen species (ROS) production and simultaneously consumes intracellular glutathione (GSH), one of the principal antioxidant defenses of cancer cells.^23^ The combined increase in oxidative stress and depletion of antioxidant capacity selectively compromises tumor cell viability because malignant cells typically contain substantially higher H_2_O_2_ levels than normal tissues, thereby favoring activation of the tellurium-based nanoprodrug system.^23^

Beyond redox modulation, recent mechanistic studies indicate that tellurium nanoparticles (TeNPs) interfere with the intracellular calcium signaling pathways. Turovsky et al. demonstrated that laser-generated TeNPs induce dose-dependent calcium responses in cancer cells and activate ROS-mediated apoptotic pathways. Increased cytosolic Ca^2+^ levels were associated with mitochondrial dysfunction, activation of apoptotic signaling cascades, and selective inhibition of cancer-cell viability, while also reducing cell migration in several tumor cell lines.^8^

In the context of precision medicine, nanotechnology enables the development of delivery systems that improve the local accumulation of tellurium nanomaterials while reducing systemic exposure. A recent example is the use of dissolvable microneedles coloaded with tellurium nanoparticles and fucoidan for melanoma therapy.^24^ In this platform, TeNPs function as photothermal agents under near-infrared (NIR) irradiation, whereas fucoidan contributes to antiangiogenic activity by inhibiting endothelial-cell migration and vascular development. The combination resulted in significant suppression of melanoma growth and reduced tumor vascular density in vivo.^24^

For bone cancer treatment, tellurium-doped mesoporous bioactive glass nanoparticles (Te-MBGs) have emerged as multifunctional therapeutic materials.^25^ These nanoparticles promote ROS-mediated apoptosis in osteosarcoma cells while preserving the mineralization capacity and bioactivity required for bone regeneration. In addition, Te-MBGs exhibit intrinsic antibacterial activity, which may help reduce the risk of implant-associated infections following surgical treatment of bone tumors.^25^ The simultaneous anticancer, antibacterial, and bone-regenerative properties of these materials highlight the versatility of tellurium-based nanoplatforms for biomedical applications.

## 5 Antimicrobial Properties and Bioactivity

The antimicrobial activity of tellurium-based nanomaterials has attracted considerable attention due to their effectiveness against clinically relevant microorganisms, including multidrug-resistant pathogens. However, the biological activity and safety profile of tellurium strongly depend on its chemical form, particle size, morphology, surface chemistry, concentration, and route of exposure. While soluble tellurium species such as tellurite (TeO3^2–^) are recognized for their toxicity, several studies have demonstrated that elemental tellurium nanoparticles (TeNPs) may exhibit antimicrobial activity at concentrations tolerated by mammalian cells under specific experimental conditions. Therefore, statements regarding safety should be interpreted within the context of the particular nanomaterial, biological model, and exposure conditions evaluated.

Several antimicrobial mechanisms have been proposed for tellurium-based nanomaterials, although their relative contribution varies according to nanoparticle composition and morphology. Hybrid tellurium–lignin nanoparticles (TeLigNPs), for example, exhibited potent antibacterial activity against *Escherichia coli* and *Pseudomonas aeruginosa*, achieving reductions greater than 5 log units while maintaining high viability of human fibroblasts and keratinocytes. Mechanistic studies demonstrated that these nanoparticles disrupt bacterial model membranes and induce intracellular reactive oxygen species (ROS) generation in Gram-negative bacteria, suggesting a combined membrane-damaging and oxidative stress-mediated mode of action.^11^ The principal antimicrobial and antibiofilm mechanisms proposed for tellurium nanoplatforms are illustrated in Figure 3.

**Figure 3:**
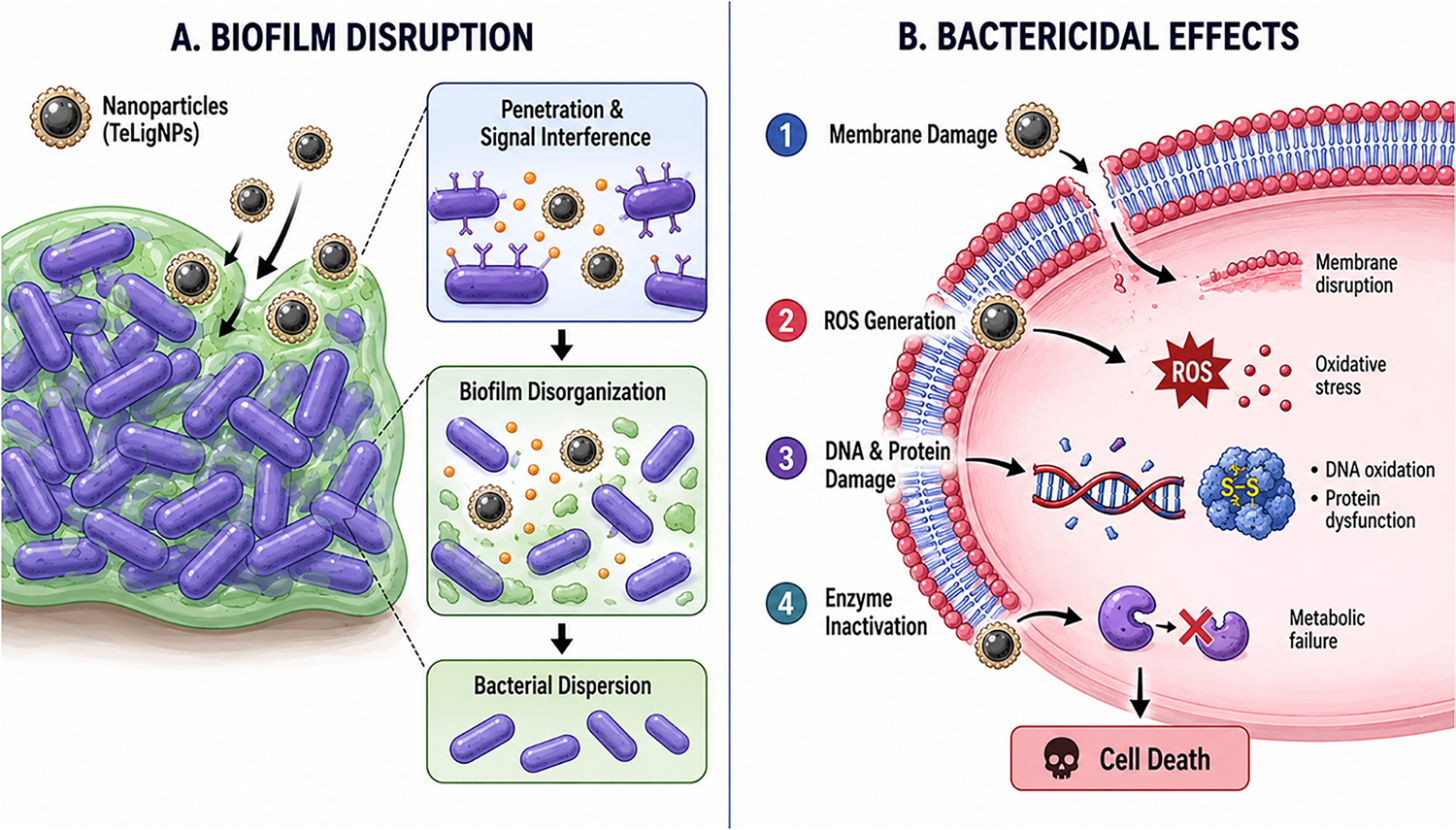
Bactericidal mechanisms and biofilm disruption by tellurium nanoplatforms. (A) Tellurium-based nanoparticles (TeLigNPs) penetrate the extracellular polymeric substance (EPS) matrix of bacterial biofilms, interfere with quorum-sensing communication, and disrupt biofilm structural integrity, leading to matrix degradation, biofilm disorganization, and bacterial dispersion. (B) Multiple bactericidal mechanisms against multidrug-resistant bacteria, including membrane disruption, intracellular ROS overproduction, oxidative damage to lipids, DNA, and proteins, thiol-mediated enzyme inactivation, metabolic failure, and subsequent cell death.

In contrast, rod-shaped biogenic tellurium nanoparticles (BioTe) appear to exert their antibacterial activity primarily through membrane damage. Tang et al. demonstrated that BioTe induced membrane perforation, increased permeability, cytoplasmic leakage, and extensive morphological damage in *E. coli*.^26^ In this system, membrane disruption was identified as the principal mechanism of antibacterial activity, whereas ROS generation and DNA damage appeared to play secondary roles. The authors proposed that positively charged nanorods initially interact with the bacterial surface through electrostatic attraction and subsequently penetrate the membrane using their sharp ends.^26^

Tellurium-containing hybrid nanomaterials have also shown promising activity against resistant pathogens. Selenium–tellurium nanoparticles (SeTeNPs) demonstrated complete inhibition of methicillin-resistant *Staphylococcus aureus* (MRSA) growth at the minimum inhibitory concentration in vitro. Fluorescence microscopy and scanning electron microscopy revealed extensive membrane damage, loss of membrane integrity, and increased bacterial cell death. Furthermore, in a bovine mastitis model caused by MRSA, intramammary administration of SeTeNPs significantly reduced bacterial counts 3 days after treatment without inducing relevant alterations in blood biochemical parameters, inflammatory markers, or organ histopathology. These findings suggest favorable biocompatibility under the experimental conditions evaluated.^27^

Biofilm-associated infections represent one of the most challenging forms of microbial resistance because extracellular polymeric matrixes protect microorganisms from antimicrobial agents and host immune responses. Hybrid TeLigNPs have demonstrated the ability to penetrate and disrupt biofilms formed by *P. aeruginosa* and *E. coli*. However, although significant biofilm degradation and reduced bacterial viability were experimentally demonstrated, the precise molecular mechanisms responsible for these effects, including possible interference with quorum-sensing pathways, remain to be fully elucidated.^11^

Collectively, current evidence supports the antimicrobial potential of tellurium-based nanomaterials through mechanisms involving membrane disruption, oxidative stress generation, and interference with essential microbial physiological processes. The main antimicrobial platforms discussed in this section, together with their mechanisms of action and available toxicity data, are summarized in Table 1. Nevertheless, these mechanisms should not be generalized to all TeNP formulations because biological activity is strongly influenced by nanoparticle composition, morphology, synthesis route, and surface characteristics. Further studies are required to establish structure–activity relationships, evaluate long-term safety, and determine the translational potential of these materials for clinical applications.

**Table 1:** Representative Antimicrobial Activities, Mechanisms, and Toxicity Profiles of Tellurium-Based Nanomaterials^a^

tellurium nanomaterial	target microorganism	antimicrobial activity	mechanism experimentally supported	toxicity evaluation
Hybrid tellurium–lignin nanoparticles (TeLigNPs)^11^	*E. coli*, *P. aeruginosa*	*>5 log reduction	Membrane disruption and ROS generation	No significant toxicity toward human fibroblasts and keratinocytes
Rod-shaped biogenic tellurium nanoparticles (BioTe)^26^	*E. coli*	**MIC = 0.78 μg/mL	Membrane perforation, increased permeability, and cytoplasmic leakage; ROS secondary	Not evaluated in mammalian cells
Selenium–tellurium nanoparticles (SeTeNPs)^27^	MRSA	Complete growth inhibition at MIC (149.70/263.95 mg/L Se/Te)	Membrane disruption, loss of membrane integrity, ROS-mediated damage (proposed)	HaCaT viability >50% at highest concentration; no significant systemic toxicity in bovine mastitis model
Biogenic tellurium nanoparticles^28^	*E. coli*, *P. aeruginosa*, *S. aureus*, *Candida albicans*	Activity observed at 500–1000 μg/mL	Not fully elucidated	Lower cytotoxicity than biogenic SeNPs in tested models

a **Note:** *>5 log reduction corresponds to a reduction greater than 99.999% of the initial microbial population. **MIC (minimum inhibitory concentration) is the lowest concentration of an antimicrobial agent capable of inhibiting the visible growth of a microorganism. Log reduction refers to the logarithmic decrease in the number of viable microorganisms.

## 6 Tellurium Toxicity and Metabolism

Although tellurium compounds have demonstrated promising applications in immunomodulation, cancer therapy, antimicrobial technologies, and nanomedicine, a critical challenge for their clinical and technological translation remains: the understanding of their toxicological behavior and metabolic fate in biological systems. Unlike selenium, which is an essential trace element involved in several antioxidant and redox-regulating enzymes, tellurium is generally regarded as a nonessential metalloid whose biological effects remain incompletely understood.^29^ Available evidence indicates that both toxicity and biocompatibility are strongly dependent on chemical speciation, oxidation state, dose, route of administration, and physicochemical characteristics of tellurium-containing materials.

Tellurium may occur in biological environments in several oxidation states, including telluride [Te­(−II)], elemental tellurium [Te(0)], tellurite [Te­(IV)], and tellurate [Te­(VI)]. Among these species, tellurite [Te­(IV)] is generally considered one of the most reactive and toxic forms because of its high affinity for thiol-containing biomolecules and its ability to disrupt cellular redox homeostasis.^29^ Following exposure, inorganic tellurium compounds undergo a series of reduction, methylation, and biotransformation processes that generate organotellurium metabolites with distinct biological properties.

Speciation studies have demonstrated that absorbed tellurium can be converted into methylated metabolites such as dimethyl telluride and trimethyltelluronium. Trimethyltelluronium is frequently detected in urine and is considered to be one of the principal terminal excretion products of tellurium metabolism. Dimethyl telluride and related volatile species may also be formed and eliminated through exhaled air, contributing to the characteristic garlic-like odor that is observed in cases of tellurium exposure. The presence of methylated tellurium species in blood and excretory fluids supports the occurrence of extensive metabolic processing after absorption.^29^ An overview of tellurium biotransformation, excretion pathways, and associated toxicological mechanisms is presented in Figure 4.

**Figure 4:**
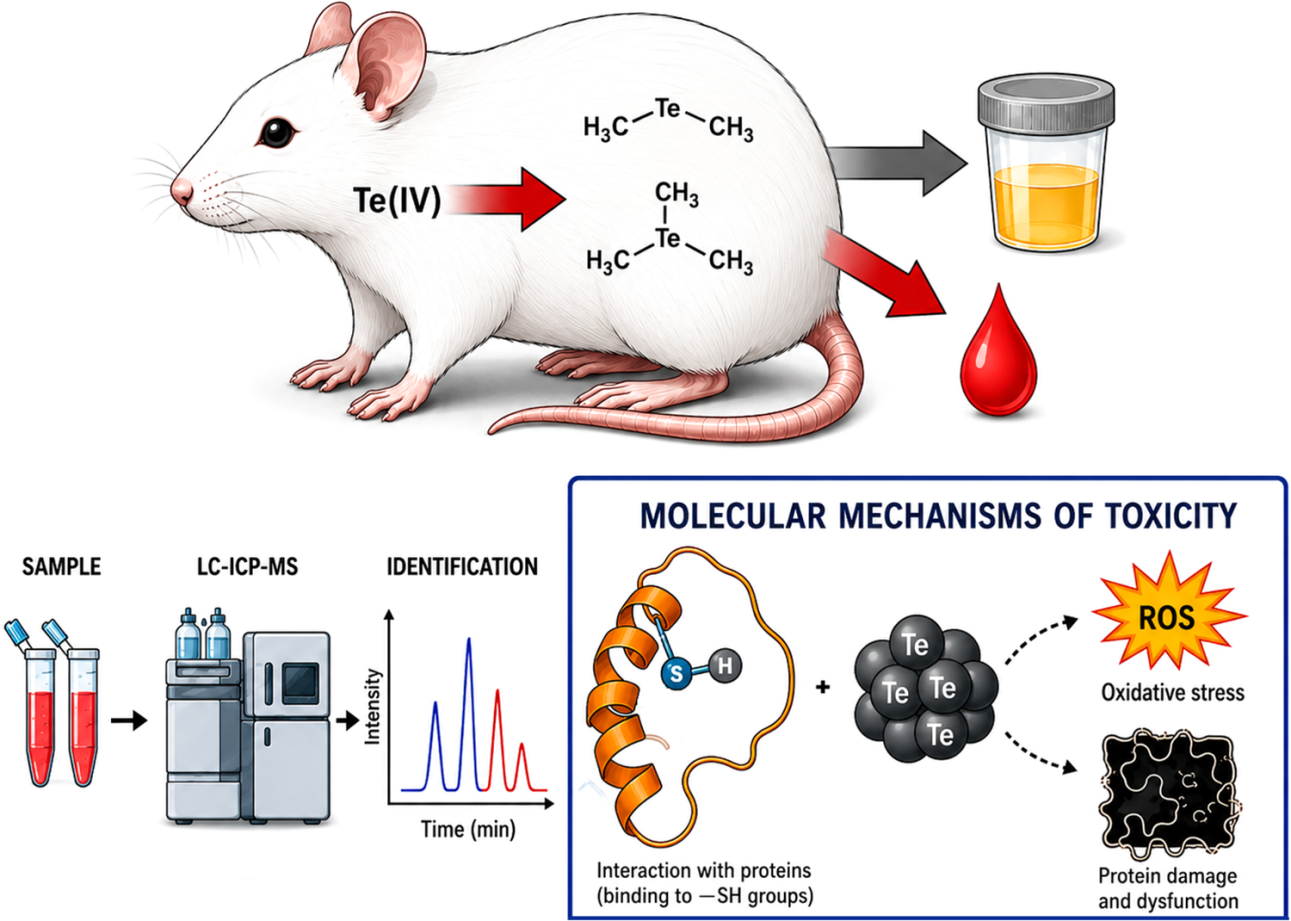
Metabolism and toxicological mechanisms of tellurium. Schematic representation of tellurite [Te­(IV)] biotransformation through reduction and methylation pathways, resulting in the formation of dimethyl telluride and trimethyltelluronium. Blood and urine are depicted as biological samples used for tellurium speciation studies by high-performance liquid chromatography coupled with inductively coupled plasma mass spectrometry (LC–ICP–MS). The proposed toxicological mechanism highlights the generation of reactive oxygen species (ROS) and subsequent protein denaturation.

At the molecular level, the toxicity of tellurium compounds is commonly associated with their interaction with sulfhydryl-containing molecules. Tellurium species readily react with protein thiols, glutathione, cysteine residues, and other redox-active biomolecules, potentially altering the enzymatic activity and disrupting cellular signaling pathways. Such interactions may contribute to oxidative stress through depletion of intracellular thiol pools, impairment of antioxidant defenses, and increased formation of reactive oxygen species.^30^ These mechanisms have been particularly described for inorganic tellurium species and several organotellurium compounds, although their relative contributions may vary according to the chemical structure and bioavailability of each compound.^30^

Importantly, the toxicological profile of tellurium-containing materials is highly dependent on their physicochemical form. Inorganic tellurium salts, especially tellurite, generally exhibit greater intrinsic reactivity and toxicity than elemental tellurium.^29^ Organotellurium compounds may display both therapeutic and toxic effects through similar thiol-reactive mechanisms, with outcomes depending on molecular structure, stability, and dose.^30^ In contrast, elemental tellurium nanomaterials have frequently shown relatively favorable biocompatibility in preclinical studies; however, their long-term biodistribution, biodegradation, tissue accumulation, and chronic toxicity remain insufficiently characterized. Likewise, hybrid Se–Te nanostructures and tellurium-doped biomaterials represent emerging platforms for which systematic toxicological evaluations are still scarce. Consequently, current evidence is insufficient to establish generalized safety conclusions for all tellurium-based systems.

The investigation of tellurium metabolism relies heavily on analytical methods capable of distinguishing individual chemical species. In this context, chemical speciation approaches, particularly high-performance liquid chromatography coupled to inductively coupled plasma mass spectrometry (LC–ICP–MS), have become essential tools for identifying and quantifying tellurium-containing metabolites in biological samples. These methodologies have significantly improved our understanding of tellurium biotransformation pathways and continue to provide critical information regarding its toxicokinetics and biological fate.^29^

Overall, the available evidence indicates that tellurium toxicity cannot be generalized across all chemical forms of the element. While several tellurium-based compounds and nanomaterials have demonstrated encouraging therapeutic potential throughout the literature, comprehensive studies addressing pharmacokinetics, biodistribution, metabolism, dose–response relationships, long-term safety, and environmental impacts remain necessary before definitive conclusions regarding their clinical safety can be established.^29,30^

## 7 Conclusion and Perspectives

The trajectory of tellurium in biomedicine has evolved significantly, moving from being viewed primarily as a potentially toxic element to becoming a versatile platform for the development of innovative therapeutic strategies. The transition from small organotellurium molecules, exemplified by AS101, to engineered tellurium nanoparticles (TeNPs) and multifunctional heterostructures has expanded the scope of biomedical applications, particularly in oncology, infectious diseases, and neurological disorders.

In oncology, tellurium-based systems have demonstrated promising therapeutic and diagnostic capabilities. Experimental studies indicate that Te-containing compounds may induce tumor cell death through mechanisms involving the generation of reactive oxygen species (ROS), disruption of intracellular calcium homeostasis, glutathione depletion, and activation of apoptosis-related pathways. Furthermore, AS101 exhibits immunomodulatory activity that may enhance antitumor responses by modulating cytokine production, regulating integrin-mediated signaling, and reducing PD-L1 expression, thereby potentially improving the efficacy of immune checkpoint therapies.^4^

Beyond cancer treatment, tellurium nanomaterials have demonstrated antimicrobial activity against clinically relevant pathogens including multidrug-resistant bacteria and biofilm-forming species. These effects have been associated with membrane disruption, oxidative stress induction, and interference with bacterial metabolic processes. In the neurological field, preclinical studies with AS101 suggest potential antidepressant and neuroprotective effects associated with modulation of brain-derived neurotrophic factor (BDNF) and neuroimmune pathways, although these findings remain limited to animal models and require further validation.^6^

Despite these advances, several challenges must be addressed before a broader clinical translation can be achieved. Future studies should establish standardized toxicological protocols capable of distinguishing the safety profiles of inorganic tellurite/tellurate salts, organotellurium compounds, elemental TeNPs, hybrid Te-based nanomaterials, and tellurium-containing biomaterials. Comprehensive pharmacokinetic, biodistribution, metabolism, and long-term toxicity studies remain necessary to support clinical development.

From a nanotechnology perspective, increasing attention has been directed toward multifunctional heterostructures incorporating tellurium with other elements, such as selenium, palladium, and gold. These systems can integrate complementary therapeutic modalities, including photothermal therapy, radiotherapy, immunomodulation, and controlled drug delivery, potentially enhancing therapeutic efficacy while reducing systemic toxicity. For example, Te–Pd–Au heterostructures have demonstrated the ability to combine radiophotothermal effects with activation of antitumor immune responses in preclinical models.^10^

Emerging delivery technologies, including stimuli-responsive hydrogels, microneedle-based platforms, and targeted nanocarriers, may further improve tissue selectivity and reduce off-target effects. Additionally, the immunomodulatory properties of certain tellurium compounds suggest potential applications beyond oncology, including chronic inflammatory and autoimmune disorders, although the evidence in these areas remains preliminary.

In summary, tellurium-based materials represent a rapidly evolving research field at the interface of coordination chemistry, nanotechnology, and biomedicine. Current preclinical evidence supports their potential as multifunctional therapeutic platforms; however, significant challenges related to safety standardization, mechanistic understanding, manufacturing reproducibility, and clinical validation remain. Continued interdisciplinary research will be essential to determining whether these promising experimental systems can ultimately be translated into safe and effective clinical applications.
